# Nurses’ evaluation of physicians’ non-clinical performance in emergency departments: advantages, disadvantages and lessons learned

**DOI:** 10.1186/s12913-015-0733-3

**Published:** 2015-02-27

**Authors:** Mohamad Alameddine, Afif Mufarrij, Miriam Saliba, Yara Mourad, Rima Jabbour, Eveline Hitti

**Affiliations:** Faculty of Health Sciences, Department of Health Management and Policy, American University of Beirut (AUB), Riad El-Solh, 1107 2020 Beirut, Lebanon; Department of Emergency Medicine, Faculty of Medicine, American University of Beirut Medical Center (AUBMC), Riad El-Solh, 1107 2020 Beirut, Lebanon; Nursing Administration, Department of Emergency Medicine, AUBMC, Riad El-Solh, 1107 2020 Beirut, Lebanon

**Keywords:** Peer evaluation, Nurses, Physicians, Emergency Department, Lebanon

## Abstract

**Background:**

Peer evaluation is increasingly used as a method to assess physicians’ interpersonal and communication skills. We report on experience with soliciting registered nurses’ feedback on physicians’ non-clinical performance in the ED of a large academic medical center in Lebanon.

**Methods:**

We utilized a secondary analysis of a de-identified database of ED nurses’ assessment of physicians’ non-clinical performance coupled with an evaluation of interventions carried out as a result of this evaluation. The database was compiled as part of quality/performance improvement initiatives using a cross-sectional design to survey registered nurses working at the ED. The survey instrument included open ended and closed ended questions assessing physicians’ communication, professionalism and leadership skills. Three episodes of evaluation were carried out over an 18 month period. Physicians were provided with a communication training carried out after the first cycle of evaluation and a detailed feedback on their assessment by nurses after each evaluation cycle. A paired *t*-test was carried out to compare mean evaluation scores between the three cycles of evaluation. Thematic analysis of nurses’ qualitative comments was carried out.

**Results:**

A statistically significant increase in the averages of skills was observed between the first and second evaluations, followed by a significant decrease in the averages of the three skills between the second and third evaluations. Personalized feedback to ED physicians and communication training initially contributed to a significant positive impact on improving ED physicians’ non-clinical skills as perceived by the ED nurses. Yet, gains achieved were lost upon reaching the third cycle of evaluation. However, the thematic analysis of the nurses’ qualitative responses portrays a decrease in concerns across the various dimensions of non-clinical performance.

**Conclusions:**

Nurses’ evaluation of the non-clinical performance of physicians has the potential of improving communication, professionalism and leadership skills amongst physicians. For improvement to be realized in a sustainable manner, such programs may need to be offered in a staged and incremental manner over a long period of time with proper dedication of resources and timely monitoring and evaluation of outcomes. Department directors need to be trained on providing peer evaluation feedback in a constructive manner.

## Background

The drive for quality, safety and accountability has historically focused the attention of health systems and professional organizations on the assessment and improvement of physicians’ clinical performance [1,2]. However, as the clinical care settings become more complex and demanding, effective communication and interpersonal skills surface as essential professional skills that physicians should master. Recent scholarly literature and professional standards have been underscoring the importance of such “soft” skills for physicians, along with sufficient medical knowledge and clinical skills [3-9].

Such changes have been evident in initiatives taken by the Accreditation Council for Graduate Medical Education (ACGME), in which physicians’ core competencies were expanded to include six main areas: medical knowledge, patient care, practice-based learning and improvement, interpersonal and communication skills, professionalism, and systems-based practice [10]. This broadening in the concept of competence has been driven by much evidence linking deficiency in the aforementioned non-clinical skills to medical errors and patient harm [3,8,11]. Furthermore, competency in these skills becomes particularly important for Emergency Department (ED) physicians due to the nature of the job and the intensity of the work environment [5,12]. More so, teamwork training has proven effective in reducing clinical errors within the ED setting [12]. For the purpose of this study, and guided by ACGME’s requirements for graduate medical education in Emergency Medicine, physicians’ non-clinical or nontechnical performance refers to mastery of three main skills: communication, professionalism, and system-based practice/leadership [10,13]. “Communication skills” entail effective communication with patients and their families, as well as other members of the health care team. “Professionalism skills” include the ability to show compassion, responsiveness, respect, accountability openness and sensitivity to patients, their families, the public and other members of the health care team. “System-based practice” entails displaying leadership in delivering health care, working inter-professionally and coordinating patient care with other members of the health care team, as well as ability to identify and rectify system issues and resolving workplace conflict [10,13].

Individual scores of medical knowledge examinations are no longer considered sufficient predictors of “soft”, i.e. non-clinical, skills including communication skills [6,14]. In fact, assessing such skills through peer ratings is a better mean to provide practical evaluation of performance in domains which cannot be assessed reliably with other tools [14]. Indeed, valuable data can be collected when peers and coworkers observe physicians, particularly regarding interpersonal and communication skills, professionalism, and certain aspects of patient care [10].

Peer assessment methods aimed at examining an individual’s work offer a more inclusive reflection of actual performance; feedback is solicited from multiple parties including those at the same level in the organizational chart, those above, and those at lower levels [10,15,16]. Such an assessment could help support employee decision making and quality improvement [10]. It also provides care providers with a synopsis of the way others perceive their performance based on their behavior at work, thus offering them the opportunity to reflect on their own conduct and compare self-perception with peer perception. Such a method of evaluation supports the identification of individuals’ strengths and weaknesses, assesses professional relations, improves clinical performance and helps improve their communication approaches with other members of the health care delivery team [6,17-21].

Despite the widespread use of peer evaluation, there remain a number of concerns about its implementation and its validity [16]. For example, some individuals may not change behavior or improve performance after receiving peer feedback [22], and others may be disheartened upon receiving negative feedback which may result in a negative reaction [6]. In addition, the existence of an authority gradient and status differentiation amongst different types of health care providers might not only affect the quality of patient care but also hinder or bias the peer evaluation process at some institutions [23]. Such findings underscore the importance of scrutinizing the process for soliciting peer evaluations, the means through which feedback was provided and the particular actions taken to act on the outcomes of such evaluations.

In the Middle East Region, peer evaluations of physicians’ non-clinical performance is a novel assessment method, as there is deficient knowledge and experience in human performance management [24]. Health care managers and decision makers need to investigate approaches to integrate peer assessment within performance improvement initiatives, while maintaining a healthy and collaborative work environment. In this study, we report on our experience with soliciting registered nurses’ feedback on physicians’ non-clinical performance in the ED setting of a large academic medical center in Lebanon.

## Methods

The study utilized a secondary analysis of a de-identified database of ED nurses’ assessment of physicians’ non-clinical performance coupled with an evaluation of interventions carried out as a result of this evaluation. The database was compiled by the institution as part of quality/performance improvement initiatives using a cross-sectional design to survey registered nurses working at the ED. In addition, and to support the analysis, a de-identified list of all physicians working in the ED with basic characteristics was obtained from the ED administration. Since the study employs a secondary analysis of a de-identified data set it was not considered human subject research and was exempt from review by the institutional review board at American University of Beirut. The study was guided by the following research questions:

- What are the advantages and disadvantages of using nurses’ assessment of physicians’ non-clinical performance in the ED setting?
- What have the research team learned in terms of enhancing chances of success and sustainability of remedial interventions?

The setting was the ED of one of the largest academic medical centers in Lebanon employing 32 ED physicians and 82 nursing staff, among which 42 are registered nurses. Three episodes of evaluation were carried out over an 18 month period.

### Survey instrument

The design of the survey instrument utilized to collect nurses’ evaluation of physicians’ non-clinical performance went through multiple phases starting with a draft that incorporated questions from a review of the literature [10,14,18]. Questions included in the survey were mainly guided by:

1. ACGME’s non-technical requirements for graduate medical education,
2. A selective review of similar tools documented in literature,
3. Complaints received from patients and health care workers,
4. ED management’s knowledge and awareness of existing problems within the institution/organization.

An expert panel, including the Director of Clinical Operations, the Director of Quality, the Nurse Manager, the ED Chairperson and an expert in performance improvement, reviewed the draft questionnaire for content validity and applicability to the given context, as well as alignment with ACGME competencies. Upon extensive deliberations, and guided by previous experiences in the organization, the group elected to adopt a short and concise questionnaire representing the three areas: communication (two questions), professionalism (six questions) and leadership (four questions). Note that leadership was used to indicate system-based approach in this manuscript. There was no perceived need to train respondents on how to take the survey as questions were arranged in a simple format, and sufficient instructions were provided on how to respond to the web-based survey. Questions asked nurses to rate physicians’ non-clinical performance on a five-point Likert scale (with clear instructions that “1” is the lowest representing poor skills and “5” is the highest representing excellent skills). For example: “Based on your experience in dealing with physician X in the ED setting, how do you rate: 1- the physician’s ability to communicate with patients and families”.

The final survey was divided into two parts. A quantitative part in which nurses assess ED physicians’: 1- communication skills (including communication with patients, families and other members of the health care team), 2- professional skills (including courtesy, approachability, responsiveness, compassion, timeliness and availability), and 3- leadership (including engaging other members of the health care team, effectively managing patient care, effectiveness in resolving conflict and leadership skills in codes). This section also included an evaluation of the physicians’ communication and interpersonal skills with patients, patients’ families, and coworkers. The qualitative part of the survey included one open-ended question asking for nurses’ feedback or concerns in regards to the non-clinical performance of evaluated physicians. Prior to dissemination, the survey was pilot tested with five randomly selected nurses (subsequently excluded from the study) to ensure clarity and solicit feedback. Necessary amendments were introduced based on the outcomes of this pilot testing.

### Data collection

Surveys were administered electronically using a web-based surveying system. They were sent to ED nurses that had worked at least two years at the hospital’s ED. Nurses were asked not to complete the survey if they work, on average, less than two shifts per month with the evaluated physician. The list of nurses was given by the ED Nurse Manager. The individual nurse responses were completely anonymous with no possibility of tracing back answers to the individual respondent.

Three evaluations were performed in May 2012, January 2013 and December 2013, separating the first evaluation by 6 months from the second one, and a year and a half from the third. The initial plan was to conduct these evaluations bi-annually; however, due to the time restrictions and scarcity of human resources, it was decided to be done yearly. A total of 27, 33 and 28 nurses were asked to evaluate 20, 21 and 23 physicians for the first, second and third round of evaluation, respectively. A total of 349 (first cycle), 255 (second cycle) and 304 (third cycle) individual nurse responses were received. The response rates were 77.7%, 46.3% and 53.6%, respectively, taking into consideration all filled surveys from the total number of surveys sent. Nurses were given up to four weeks to complete the questionnaire and were reminded via email twice during that period. It is worth mentioning that 17 physicians were evaluated in all three rounds of evaluation.

**Figure 1:**
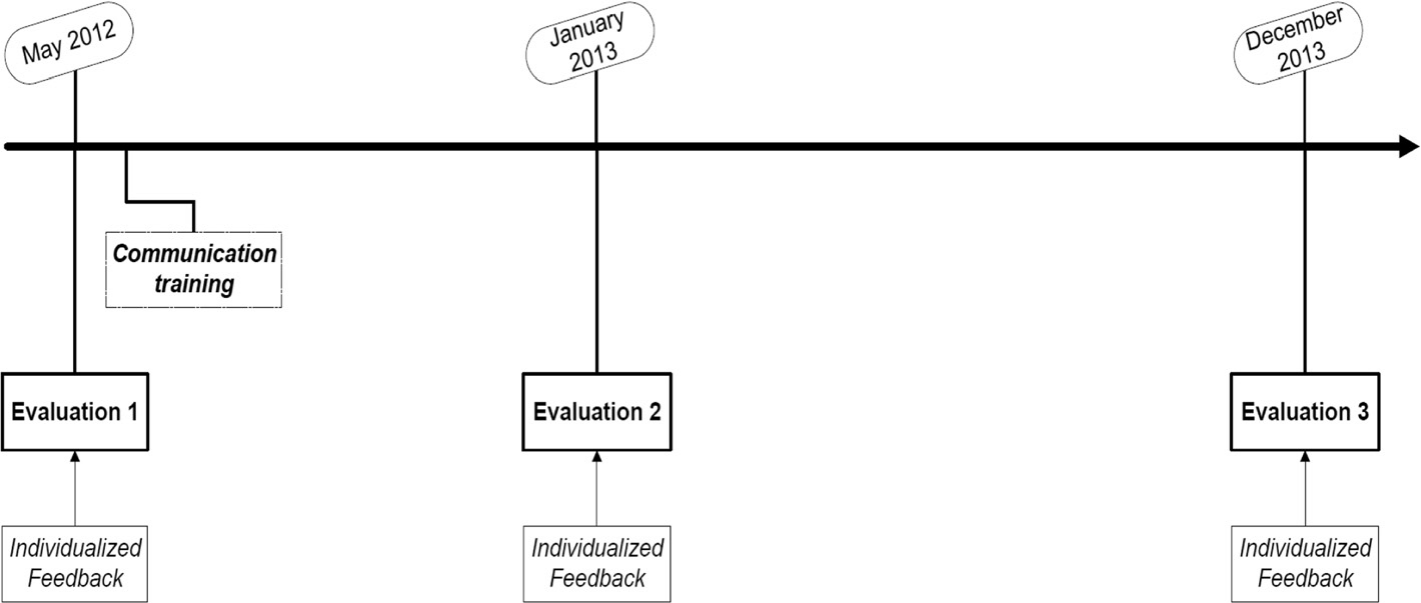
**The timeline of evaluations and interventions carried out.**

Specific interventions took place after each cycle of evaluation (Figure 1). The chosen interventions have been documented in literature [3,10,20,25]. In addition, these were the interventions deemed most appropriate to the process improvement expert panel within the boundaries of time and money. Finally, the interventions have been endorsed and well received by both physicians and nurses at the ED. The interventions included:

1. Providing ED physicians with a detailed feedback on the assessment of nurses (with no identifiers) of their non-clinical performance: Sharing peer assessment feedback has been shown to be helpful and has reportedly been associated with favorable transformations in performance [10,20]. Each of the ED physicians was invited to a personal meeting with the Chairperson of the ED and was provided with an anonymous summary of the nurses’ evaluation of his/her performance. In all cycles, the Chairperson of the ED reviewed that metric as well as the nursing comments in person with the individual physician, listened to the physician’s feedback, reviewed expectations and developed an action plan for improvement with the physician. Prior to sharing the evaluations with the individual physician, the Chairperson of the ED and the Director of Professional Practice Development reviewed the evaluations and removed all derogatory/strongly offensive language from the open-ended text. The quantitative analysis was included as a performance metric within the ongoing physician performance evaluation record while the additional comments were placed on a separate sheet that was not part of the permanent record. The expert panel and the research team concurred that deleting offensive and derogatory comments and placing qualitative comments outside physicians’ performance records would help avoid insulting physicians which could reflect negatively on the work environment and would contradict the purpose of this intervention.
2. A communication training workshop carried out after the first cycle of evaluation: Literature has shown that such interventions that incorporate communication skills training have resulted in positive impacts, and acquired skills have been integrated into practice post intervention [3,25]. The ED hired an external consultant to deliver communication training to ED physician and nursing staff. In December, 2012, three four-hour training sessions were delivered to equally mixed groups of nurses, physicians and multi-functional technicians. The sessions covered basic communication and conflict resolution training as well as application of covered concepts through role plays of ED-based scenarios. A total 19/21 (90%) physicians and 58/69 (84%) nursing staff attended the sessions.

### Statistical analysis

#### Quantitative section

The rating scale used in the quantitative section of the survey involved five ordered response levels from 1 (poor) to 5 (excellent). A paired *t*-test was carried out to compare the mean scores of performance on communication skills, professional skills and leadership skills, between the three cycles of evaluation. Time period since training completion and duration of appointment at ED were treated as continuous variables.

#### Qualitative section

A thematic analysis was conducted for data collected in this section. The nurses’ comments were reviewed and analyzed for recurrent themes. Results were then communicated to physicians during the provision of feedback to improve physicians’ leadership and management competencies.

## Results

### Sample description

Table 1 presents a detailed description of the physicians’ personal and professional characteristics. The majority of the ED physicians are males (81.5%). As for their professional characteristics, most of these physicians are not specialized in Emergency Medicine (77.8%), are employed as part-timers (74.1%), and work during the day (70.4%). The physicians working in the ED are almost equally divided between the three sections: high acuity (29.6%), low acuity (29.6%) and mixed acuity (22.2%); with pediatrics constituting 18.5%. More than half of the evaluated physicians (55.6%) completed their post-residency/fellowship training in Lebanon. In addition, more than three quarters completed residency training after year 2000, with almost 60% completed during the past four years. As for appointment date, the majority of the physicians were appointed during the past three years (70.4%), with only 7.4% working at the ED for more than 10 years.

**Table 1:** **Absolute and relative distribution of ED physicians by selected demographic and professional characteristics (N = 27)**

**Variable**	**Categories**	**N (%)**
**Gender**	Male	22 (81.5)
	Female	5 (18.5)
**Specialty**	Emergency medicine	6 (22.2)
	Non-emergency medicine	21 (77.8)
**Working status in this ED**	Full-time	7 (25.9)
	Part-time	20 (74.1)
**Type of shift**	Day	19 (70.4)
	Night	8 (29.6)
**ED section of work**	High acuity	8 (29.6)
	Low acuity	8 (29.6)
	Mixed acuity	6 (22.2)
	Pediatrics	5 (18.5)
**Location of post-residency/fellowship training**	National	15 (55.6)
	International	12 (44.4)
**Date of training completion**	Prior to 1999	6 (22.2)
	2000–2009	5 (18.5)
	2010-2014	16 (59.3)
**Appointment date to current ED**	<1 year	3 (11.1)
	1–3 years	16 (59.3)
	4–10 years	6 (22.2)
	> 10 years	2 (7.4)

### Quantitative data analysis

A paired *t*-test was carried out to compare the mean scores of physicians’ performance across the three cycles of evaluation. As Table 2 displays, the scores in the second cycle were higher than that in the first cycle on the three dimensions of non-clinical performance (communication, professionalism and leadership skills), as well as on the overall evaluation. Yet, the difference was statistically significant only for leadership skills (P = 0.005) and overall evaluation (P = 0.001). In contrast, however, the scores in the third cycle of evaluation were significantly lower than that of the second on the three dimensions of non-clinical performance, as well as on the overall evaluation. No significant differences were found between the first and third cycles of evaluation, on the three dimensions and on the overall evaluation.

**Table 2:** **Results of the paired** ***t*** **-test comparing the mean scores of performance among the three cycles of evaluation**

**Dimension**	**Communication skills**	**Professionalism skills**	**Leadership skills**	**Overall evaluation**
**Pairs**	**Mean ± SD**	**Mean ± SD**	**Mean ± SD**	**Mean ± SD**
**Evaluation 1**	4.34 ± 0.33	4.35 ± 0.36	4.03 ± 0.47	4.24 ± 0.36
**Evaluation 2**	4.49 ± 0.49	4.52 ± 0.45	4.36 ± 0.50	4.46 ± 0.46
**p value**	0.181	0.120	**0.005**	**0.001**
**Evaluation 2**	4.49 ± 0.49	4.52 ± 0.45	4.36 ± 0.50	4.46 ± 0.46
**Evaluation 3**	4.29 ± 0.38	4.26 ± 0.34	4.03 ± 0.44	4.19 ± 0.35
**p value**	**0.001**	**0.000**	**0.000**	**0.000**
**Evaluation 1**	4.34 ± 0.33	4.35 ± 0.36	4.03 ± 0.47	4.24 ± 0.36
**Evaluation 3**	4.29 ± 0.38	4.26 ± 0.34	4.03 ± 0.44	4.19 ± 0.35
**p value**	0.723	0.201	0.373	0.702

Note: P-values less than 0.05 are considered significant and are bolded in the Table.

**Figure 2:**
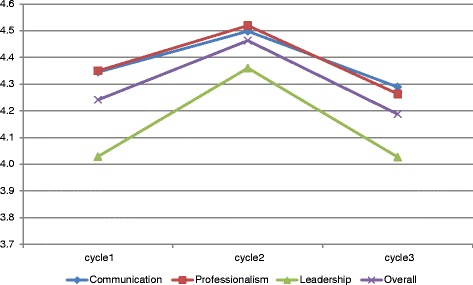
**Physicians’ change in skills between the three cycles of evaluation.**

Figure 2 provides a summary of the changes in the overall evaluation of physicians’ skills across the three evaluation cycles. As exhibited in the figure, there is a significant increase in the averages of the three skills between the first and second cycles of evaluation (during the period of the first 6 months), followed by a significant decrease in the averages of the three skills between the second and third cycles of evaluation (during the period of the following year). The same trend can be observed for the overall physicians’ averages among the three evaluations.

### Qualitative data analysis

Thematic analysis of nurses’ qualitative remarks reveals that such comments were mainly made to voice specific concerns towards one or more of the evaluated physicians. Note that very few positive comments were made by nurses and that no differences were observed in the frequency of positive comments, as per the thematic analysis, between the three cycles of evaluation.

As Table 3 portrays, the thematic analysis of the qualitative responses provided by nurses across the three cycles of evaluation exposes an overall decrease in concerns and negative comments across the various dimensions of non-clinical performance, most remarkably, across the “management of ED patients”, “courtesy and professionalism”, “approachability” and “engaging patients in plan of care” dimensions.

**Table 3:** **Summary of concerns/comments communicated by nurses in the three cycles of evaluation**

	**Dimension of performance**	**Number of written concerns/comments**		
		**First cycle**	**Second cycle**	**Third cycle**
1	Communication with patients and their families	Five	Two	Zero
2	Courtesy and professionalism	Nine	Zero	Two
3	Temper	Four	Three	One
4	Approachability	Six	One	Zero
5	Responsiveness to concerns	Five	Zero	Zero
6	Non-clinical quality of care	Six	Zero	Zero
7	Overall Management of ED patients	Fifteen	One	Three
8	Initiative and responsibility in conflict resolution	Nine	One	One
9	Availability in ED	Six	Zero	One
10	Engaging the nurses in plan of care	Seven	Zero	One

## Discussion/conclusions

### What have we learned?

Analysis of the quantitative data collected from the nurses evaluations of ED physicians revealed that evaluation scores on all three dimensions of non-clinical performance witnessed an improvement between the date of Evaluation 1 (May 2012) and Evaluation 2 (January 2013). The first cycle of evaluation served as a baseline assessment after which two interventions were introduced: individualized feedback to ED physicians and a communication training program offered to ED providers. Providing highly skilled individuals, such as ED physicians, with feedback on performance requires considerable skill and experience in human performance appraisal in order to ensure that such an exercise would result in performance improvement instead of putting the physicians on the defensive or increasing their anger with the nurses that work with them [6,10,26,27]. Engaging physicians in the implementation process of nursing-evaluation from feedback on surveys and selection of nurse eligibility criteria for participation is essential for subsequent willingness of physicians to receiving the feedback. In addition, it is highly recommended for clinical settings that would like to embark on peer evaluation exercises to train their Chairs and Directors on the appropriate skills and communication techniques that would empower them to skillfully provide their staff with transparent feedback. This is while maintaining a collaborative work environment and avoiding the aforementioned non-intended negative attitude/consequences [10,14,28].

The improvement in the evaluation scores between the first and second evaluation cycles was a temporary result, as evaluation scores during the third evaluation cycle dropped significantly, to reach close to the levels of the first evaluation cycle. Following the second evaluation, no trainings were offered and the only intervention was the offering of individualized feedback. Such findings reflect the short-lived effects that the communication training program had on the non-clinical skills of ED physicians. It appears that the intensity of work at a clinical setting in general, and that of the ED in particular, may have diluted the effects of the introduced interventions, and the acquired desired behavior may have been forgotten or overlooked. Therefore, it becomes evident that refresher/booster courses are necessary to maintain the desired change in the non-clinical skills of providers [12,29]. The sharp decline observed in the third cycle of evaluation could also be attributed to the increasing expectations of nurses for continuous improvement in the non-clinical performance of ED physicians. Clinical Directors and Chairs are encouraged to offer training programs dispersed around the year with continuous feedback from participants on challenges faced in non-clinical performance and the means for the clinical team to overcome them.

Moreover, the timeframe between the first and second evaluation cycles was six months compared to one year between the second and the third. The early reassessment may have helped maintain the performance observed from results of the second evaluation. The time effort involved in evaluating physicians every six months was, however, prohibitive to maintaining this frequency of reassessment. From a management perspective, three key lessons could be learned from this finding. First, human resources performance appraisal is an exercise that requires good planning and dedication of human and financial resources in order to be able to carry it out appropriately and assess improvements in performance, or the lack thereof, in a timely manner [30]. Second, it is imperative to dedicate resources in order to carry out individual or team-based training programs to act on the results of the assessment. Depending on the training and desired behavioral change, such programs may need to be offered in a staged and incremental manner over a long period of time [12]. Therefore, it is important to dedicate such resources in the human resources development plan of the particular department. Last, resources and expertise should also be dedicated for the proper monitoring and evaluation of the outcomes of human resources evaluation programs and associated skills and behavioral change interventions [31].

The thematic analysis of qualitative results reveals an overall decrease in nurses’ concerns and comments across many of the dimensions of performance; however, it has to be noted that the number of comments provided was small compared to the large number of participating nurses. Yet, it cannot be ascertained whether nurses’ negative comments decreased due to improved physicians’ performance or due to them refraining from providing comments because they do not trust that changes will take place. This cannot be ascertained until a formal evaluation is carried out preferably including a qualitative component involving interviews or focus group discussions with nurses.

There are a number of shortcomings in this study that are worth mentioning. First, there is a risk for participation bias since not all nurses invited to complete the peer evaluation of ED physicians agreed to participate. Therefore, it cannot be ascertained whether participating nurses would hold any significantly different views or biased opinions compared to their non-participating counterparts. Having said that, the percentage of participating nurses remains high across the three cycles of evaluation taking into consideration that this is a voluntary online evaluation questionnaire sent to exceptionally busy ED nurses. Second, despite multiple assurances of complete confidentiality by the ED management, there is a risk for social desirability bias by which nurses may have biased their answers in order to please ED physician colleagues or in fear of disclosure. Third, since the nurse identity was protected throughout the study, tracing a nurse’s response individually across the three episodes of evaluation was not feasible. Rather, the study team evaluated ED physicians’ non-clinical performance through measuring changes in the average evaluation provide by all surveyed nurses, both per physician and for all physicians. Last, the non-experimental cross-sectional nature of the study does not enable the establishment of causality between the peer evaluation process and changes in physicians’ non-clinical performance; the data rather points towards certain plausible associations.

Nevertheless, nurses’ evaluation of the non-clinical performance of physicians has the potential of improving communication, professionalism and leadership skills amongst physicians. For the effect of such programs to be realized in a sustainable manner, the Chairs of clinical departments need to be trained on providing peer evaluation feedback in a constructive and motivating manner. Additionally, behavioral change programs need to be offered over a long period of time with proper dedication of resources and timely monitoring and evaluation of changes in the attitude and behavior of clinical staff.
